# Supplementary material for: Mucin4 (MUC4) Antibody Labeled with an NIR Dye Brightly Targets Pancreatic Cancer Liver Metastases and Peritoneal Carcinomatosis
Source: Cancers (Basel). 2025 Jun 18;17(12):2031. doi: 10.3390/cancers17122031 (PMC12191291; doi:10.3390/cancers17122031)
Supplement: Supplementary file 1 [file cancers-17-02031-s001.zip › cancers-3639179-supplementary.pdf]

Article

Mucin4 (MUC4) Antibody Labeled with an NIR Dye Brightly Targets Pancreatic Cancer Liver Metastases and Peritoneal Carcinomatosis

Sunidhi Jaiswal [1,2], Siamak Amirfakhri [1,2], Javier Bravo [1,2], Keita Kobayashi [1], Abhijit Aithal [3], Sumbal Talib [4], Kavita Mallya [3], Maneesh Jain [3], Aaron M. Mohs [3,4], Robert M. Hoffman [1,2,5], Surinder K. Batra [3] and Michael Bouvet [1,2]

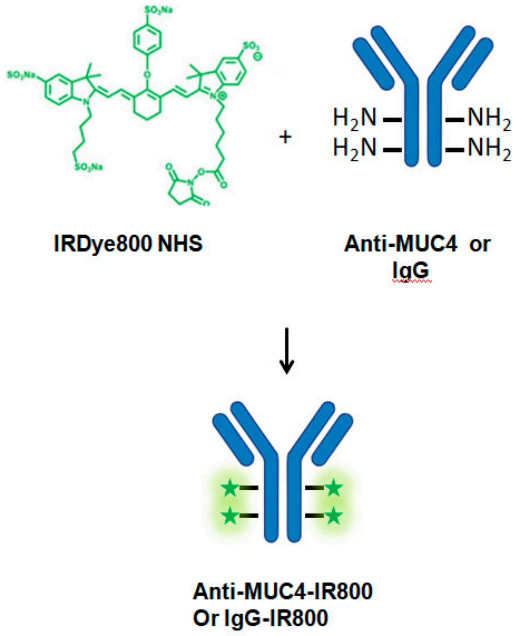

Figure S1. Schematics of labeling of anti-MUC4 antibody and IgG control antibody with IRDye800NHS.

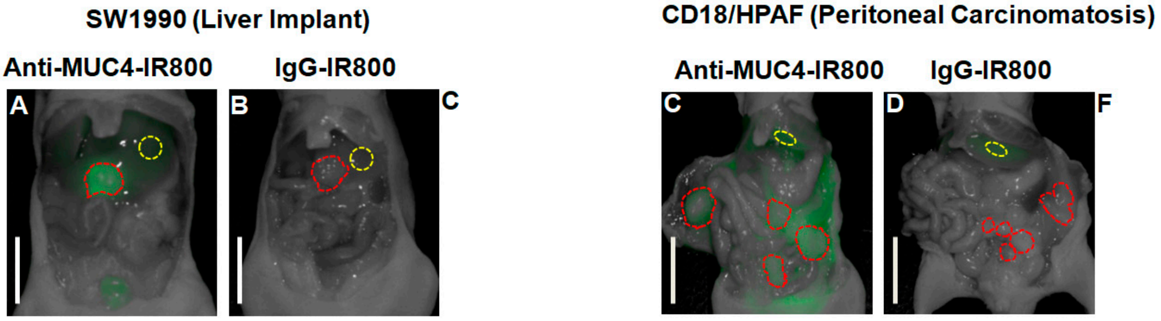

Figure S2. Determination of the region of interest in liver implant mouse models (A and B), and peritoneal carcinomatosis mouse models (C and D). Red dashed regions represent tumors and yellow dashed regions represent normal liver. Images are combined bright light and NIR images. For all images of both models were treated with either anti-MUC4-IR800 or IgG-IR800. The same brightness and contrast settings were used for both experimental and control group.
